# Neck–tongue syndrome secondary to atlantoaxial dislocation

**DOI:** 10.3389/fsurg.2026.1821255

**Published:** 2026-07-28

**Authors:** Jing Wang, Jin Zhang, Haihong Zhang

**Affiliations:** 1 Department of Orthopedics, The Second Hospital of Lanzhou University, Lanzhou, China; 2 Department of Orthopedics, Gansu Provincial Hospital of Traditional Chinese Medicine, Lanzhou, China

**Keywords:** atlantoaxial dislocation, C2 root, lateral atlantoaxial joint, neck–tongue syndrome, posterior fusion

## Abstract

**Background:**

Neck–tongue syndrome is a rare paroxysmal disorder characterised by occipito-cervical pain with ipsilateral hemilingual numbness or dysaesthesia, typically triggered by head rotation or prolonged speaking. Although abnormal mechanical stimulation of the C2 spinal nerve/ root ventral ramus near the lateral atlantoaxial joint is widely implicated, neck-tongue syndrome occurring in patients with atlantoaxial dislocation has been seldom described.

**Methods:**

We retrospectively reviewed 176 consecutive patients with atlantoaxial dislocation who underwent posterior reduction and instrumented fusion between September 2018 and October 2025. Patients fulfilling prespecified diagnostic criteria for neck-tongue syndrome were identified. Demographics, aetiology, symptom duration, pain severity assessed using the visual analogue scale, neurological status assessed using the Frankel grade, imaging findings, operative details, and postoperative outcomes were analysed.

**Results:**

Six patients met criteria for NTS, yielding a prevalence of 3.4% (6/176). Symptom duration was 3–17 months with a median of 9 months. Preoperative visual analogue scale scores ranged from 4 to 8with a mean of 5.67 ± 1.37. All patients reported reproducible tongue symptoms including pain, numbness, or dysaesthesia, provoked by head turning or sustained speaking; symptoms were alleviated by cervical immobilisation or complete neck muscle relaxation in the supine position. Two patients showed mild hemilingual atrophy with dysarthria. Four patients had limb sensory symptoms; preoperative Frankel grades were B in one patient, C in one patient, and E in four patients. All six underwent. All six underwent posterior atlantoaxial reduction and fusion; C2 root sacrifice was performed in two cases to facilitate joint release and implant placement. During follow-upof 3–63 months, with a median of 30 months, neck-tongue syndrome-related symptoms improved in all patients, with visual analogue scale scores decreasing to 0–3 at the last follow-up with a mean of 1.16 ± 1.47. Radiographs confirmed satisfactory reductiondefined as an atlantodental interval of less than 3 mm, and solid fusion. Two patients who underwent C2 root sacrifice had persistent focal occipital or scalp hypoesthesia without neurogenic ulceration or pressure sores.

**Conclusion:**

In atlantoaxial dislocation, pathological hypermobility of the lateral atlantoaxial joint may cause repeated dynamic impingement and traction on the C2 root ventral ramus, triggering tongue- related afferent disturbances and the clinical phenotype of neck-tongue syndrome. Posterior reduction and instrumented fusion can interrupt this dynamic irritation and are associated with substantial symptom relief.

## Introduction

**Figure 1:**
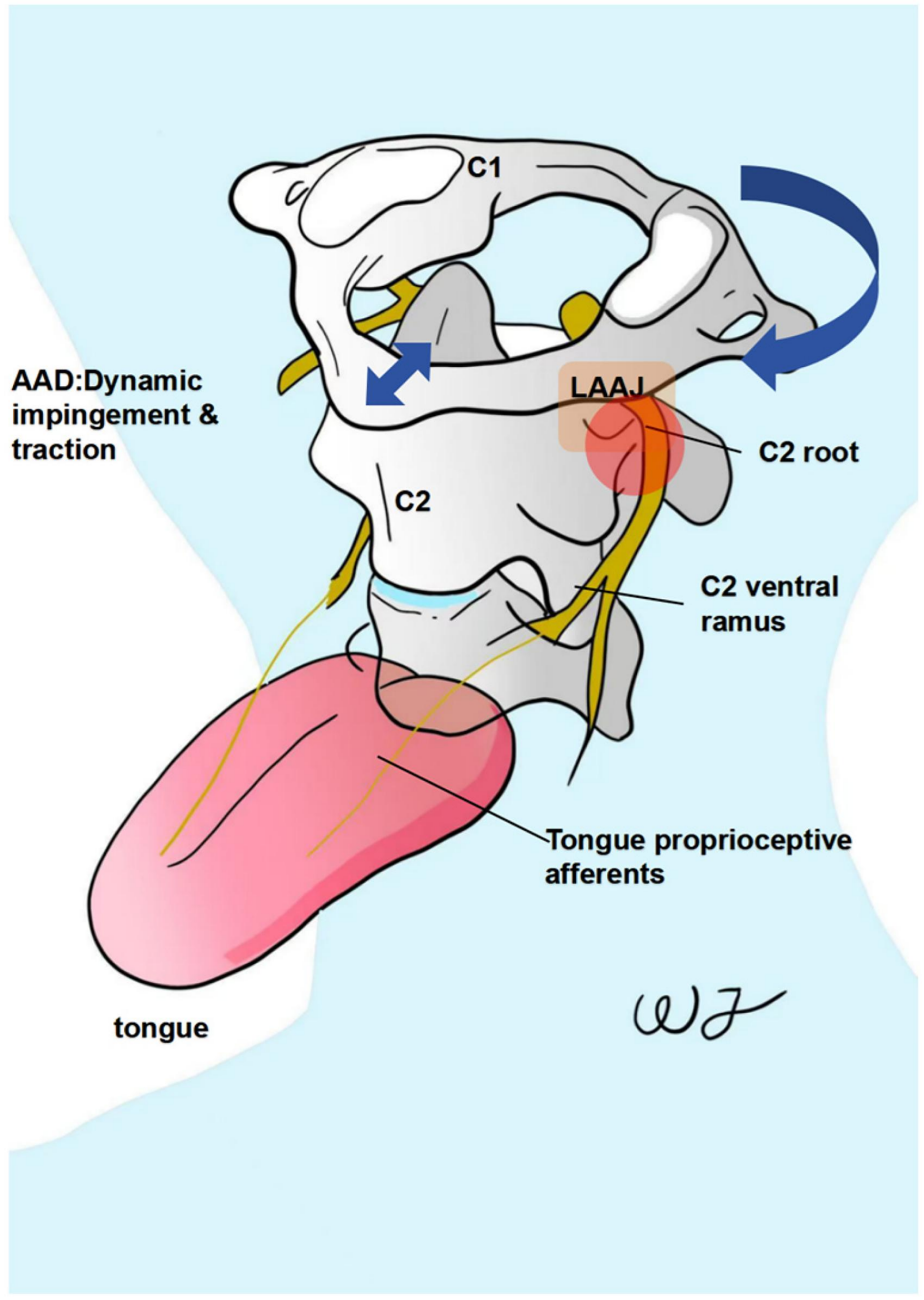
Proposed mechanism of neck–tongue syndrome secondary to atlantoaxial dislocation.this is an original schematic illustration created by the authors for this manuscript. Baseline anatomy and proximity: the C2 spinal nerve (root) and ventral ramus course near the posterior margin of the lateral atlantoaxial joint (LAAJ), allowing afferent input related to tongue sensation (via lingual afferents) to be susceptible to mechanical irritation. In AAD, dynamic instability—typically provoked by head rotation and sometimes by prolonged speaking—may cause repeated impingement and traction of the C2 root/ventral ramus at the posterior LAAJ margin, leading to occipito–cervical pain and ipsilateral hemilingual sensory symptoms.

Neck–tongue syndrome (NTS) was first described by Lance in 1980 as a distinctive paroxysmal syndrome in which sudden occipital or upper cervical pain is accompanied by ipsilateral numbness, paraesthesia, or dysaesthesia of the tongue, most commonly triggered by head rotation (1). The condition is uncommon, and its true incidence remains uncertain; reported cases include idiopathic, familial, and secondary forms (2–5), with the latter more common among women and adolescents (6–9). Despite heterogeneous aetiologies, a unifying mechanistic concept is abnormal mechanical irritation of the C2 spinal nerve or its ventral ramus in proximity to the lateral atlantoaxial joint (LAAJ) (1–6). Anatomically, the ventral ramus of the C2 spinal nerve courses adjacent to the LAAJ and gives articular branches to the joint capsule (2, 10). Sensory afferents from the tongue, particularly proprioceptive inputs conveyed via lingual pathways, have been proposed to converge on C2-related neural structures, providing a plausible substrate for tongue symptoms when the C2 nerve is stimulated near the atlantoaxial complex (8). Atlantoaxial dislocation (AAD), resulting from congenital deformity, inflammation, tumour-related destruction, trauma, or other craniovertebral junction pathology, produces pathological instability and abnormal motion at the atlantoaxial complex. Such dynamic instability may increase repetitive impingement or traction at the posterior margin of the LAAJ, potentially predisposing susceptible individuals to NTS (Figure 1).

Posterior reduction and instrumented fusion are established surgical strategies for symptomatic AAD when mechanical instability, neural compression, or failure of conservative treatment is present. In the context of AAD-associated NTS, surgical stabilisation is clinically relevant because it may eliminate the repeated motion-dependent irritation of the C2 spinal nerve root ventral ramus rather than merely suppressing pain symptoms.

**Figure 2:**
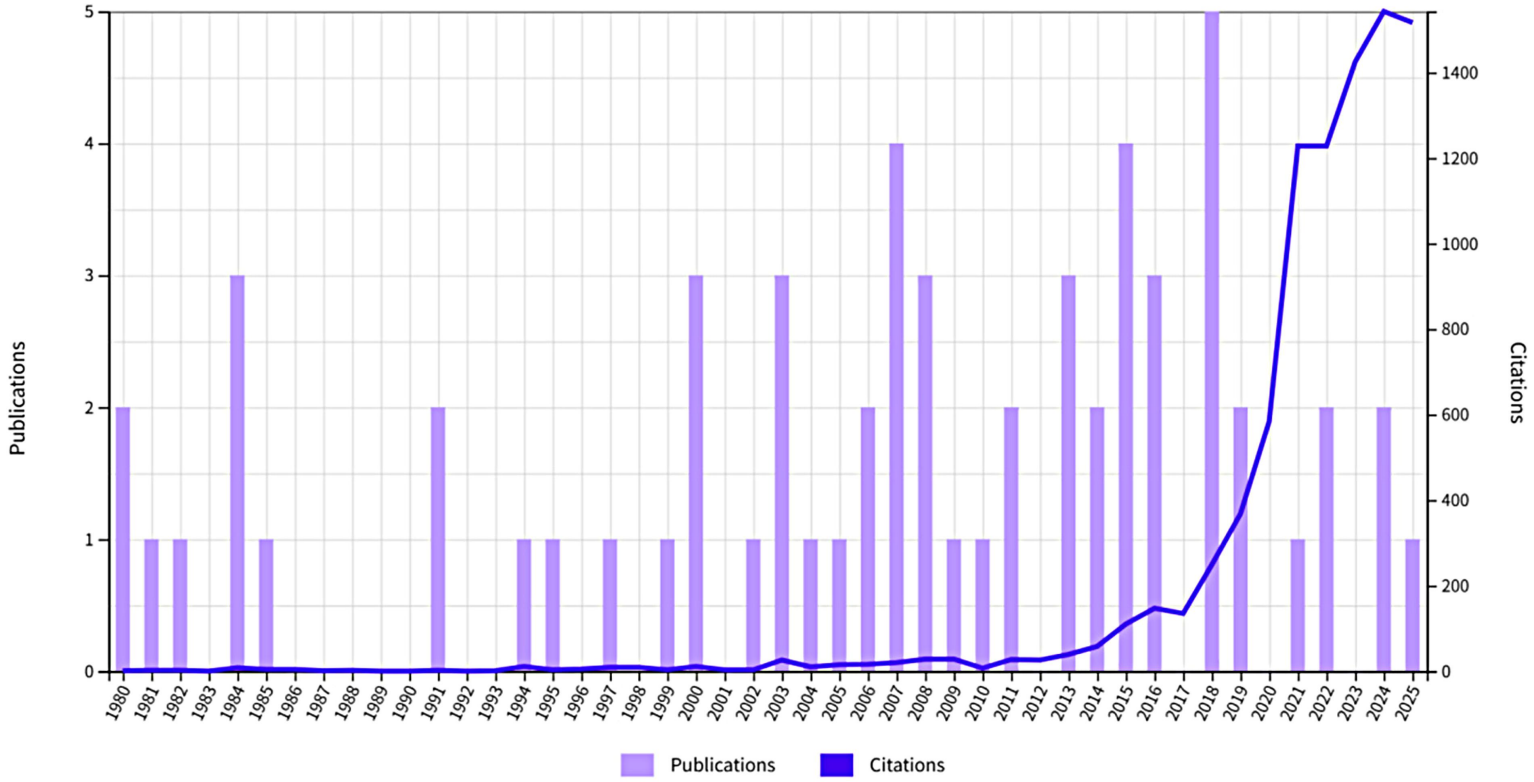
NTS publishes annual trends (1980–2025). The *X*-axis represents the years (1980–2025); the *Y*-axis represents the number of articles published; the bar chart shows the number of articles; the line graph shows the number of citations of the articles.

**Figure 3:**
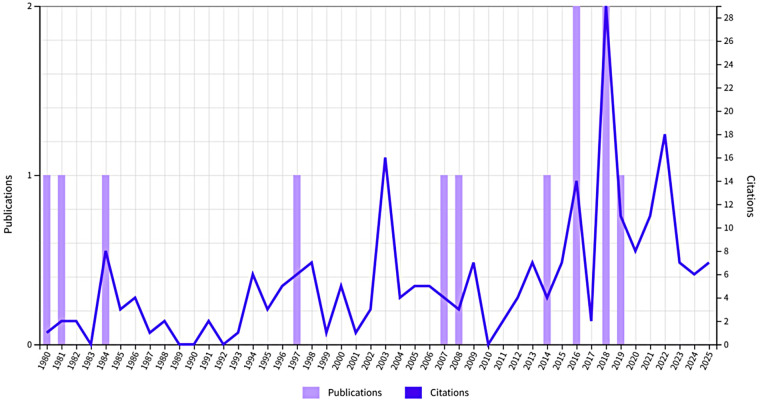
AAD + NTS publishes annual trends (1980–2025).

Most published reports of NTS involve minor cervical trauma, idiopathic presentations, or mild degenerative cervical changes (5, 7, 8, 11). In contrast, atlantoaxial dislocation (AAD), resulting from congenital anomalies, inflammation, tumours, or major trauma, produces pathological instability and abnormal motion at the atlantoaxial complex. Such dynamic instability may increase repetitive impingement or traction forces at the posterior margin of the LAAJ, potentially predisposing susceptible individuals to NTS (Figure 1). However, the prevalence and clinical features of NTS in a well- defined surgical AAD population, as well as outcomes following surgical stabilisation, remain poorly characterised. A topic search of the Web of Science All Databases (WOSAD) using (“neck-tongue syndrome” OR “neck tongue syndrome”) identified 65 records published between 1980 and December 15, 2025. After excluding review articles, 61 original articles were retained. A narrower topic search combining NTS terms with AAD-related terms-(“neck-tongue syndrome” OR “neck tongue syndrome”) AND (atlantoaxial OR “atlanto-axial” OR “atlantoaxial dislocation” OR “atlantoaxial subluxation” OR “atlantoaxial instability” OR AAD OR “lateral atlantoaxial joint” OR “Os Odontoideum”)-returned 12 records; after excluding review and non-English articles, 10 articles were included for qualitative appraisal (Figures 2, 3). These reports did not provide a systematic description of NTS secondary to surgically treated AAD. Therefore, the prevalence, clinical features, and surgical outcomes of NTS in a well-defined AAD cohort remain poorly characterised.

In this retrospective, cohort study, we report six patients with NTS identified among 176 surgically treated AAD cases, describe their demographic, etiological, clinical and radiological characteristics, and evaluate symptom response after posterior reduction and fusion.

## Materials and methods

### Design

This study was a single-center retrospective cohort study conducted in accordance with the principles of the Declaration of Helsinki. It was approved by the Medical Ethics Committee of the Second Hospital of Lanzhou University (approval No. 2024A-093). Clinical trial registration was not applicable because of the retrospective design. Written informed consent was obtained from all individual participants included in this retrospective study. We retrospectively reviewed 176 consecutive patients with who underwent posterior reduction and instrumented fusion in the Department of Orthopedics at our institution between September 2018 and October 2025. The variables collected included demographic data, etiology, preoperative AAD classification, symptom duration, NTS-related symptoms, visual analogue scale (VAS) score, Frankel grade, imaging findings, operative details, C2 root management, radiographic reduction and fusion status, complications, and follow-up duration.

## Methods

All 176 eligible patients were systematically screened for NTS symptoms after surgery. Each patient was contacted individually by telephone or WeChat and interviewed using a structured questionnaire that specifically asked about transient ipsilateral tongue numbness/paresthesia, dysesthesia, pain, or a subjective “tongue shortening' sensation precipitated by head rotation and/or prolonged speaking, with or without concomitant occipito–cervical pain, for patients who screened positive, outpatient and inpatient records were reviewed to corroborate symptom features, temporal relationships, imaging findings, and postoperative outcomes. No randomization, allocation concealment, prospective recruitment, or study grouping was performed because this was a retrospective consecutive surgical cohort.

**Figure 4:**
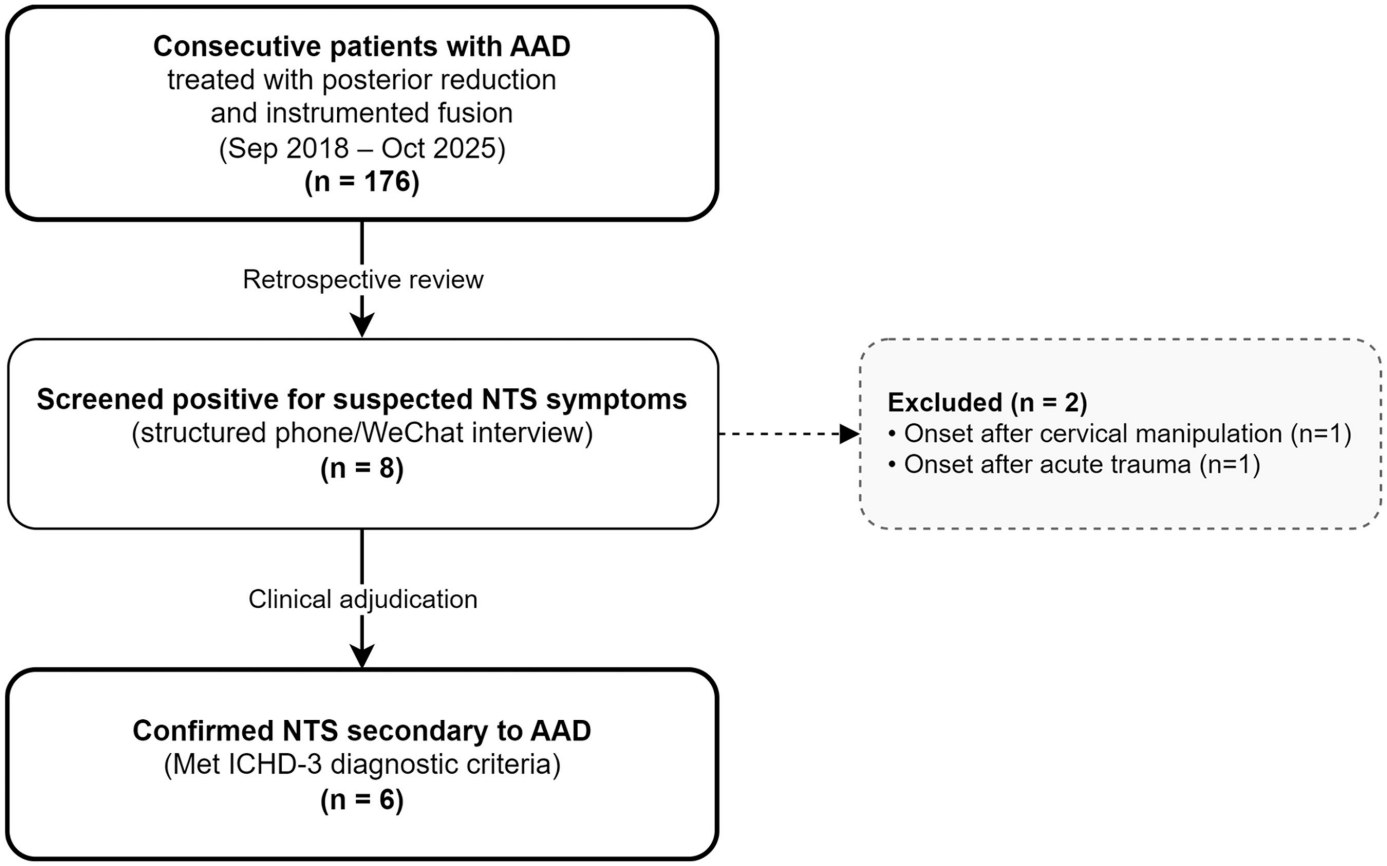
Patient selection flow. A total of 176 patients with atlantoaxial dislocation (AAD) treated with posterior reduction and instrumented fusion were screened for suspected neck–tongue syndrome (NTS) symptoms using a structured telephone/WeChat interview. Eight screened positive; two were excluded because symptoms occurred only after cervical manipulation/trauma; six patients met ICHD-3 diagnostic criteria and were included.

A diagnosis of NTS was adjudicated according to the International Classification of Headache Disorders, 3rd edition (ICHD-3) (1, 5). Patients whose tongue symptoms occurred only after cervical manipulation or acute trauma cute trauma without persistent motion-provoked symptoms attributable to AAD were excluded from the NTS cohort. Exclusion criteria included: (1) tongue symptoms without a consistent relationship to neck movement, prolonged speaking, or cervical posture; (2) NTS symptoms emerging after cervical manipulation therapy or acute neck trauma; and (3) previous surgery involving the atlantoaxial complex (Figure 4). Preoperative AAD type was classified according to Yin's clinical classification system, in which AAD is categorised into reducible AAD, irreducible AAD, and fixed AAD based on preoperative radiographs, dynamic radiographs, computed tomography, and magnetic resonance imaging (12).

### Instrumentation

Pain intensity was assessed using the VAS, and neurological function was graded using the Frankel grade preoperatively and at follow-up. Preoperative and postoperative radiographs, including anteroposterior, lateral, and flexion-extension views when available, were used to evaluate atlantoaxial alignment and dynamic instability. CT including three-dimensional reconstruction, was used to assess osseous anatomy, atlantoaxial reduction, implant position, and fusion status. MRI was reviewed to evaluate spinal cord compression and soft tissue pathology. These are standard clinical and radiological assessment tools routinely used in upper cervical spine surgery.

### Procedure

All patients underwent posterior atlantoaxial reduction and instrumented fusion under general anaesthesia. The fixation form (C1- C2 fusion or occipitocervical fusion) was selected according to underlying pathology, craniovertebral anatomy and intraoperative stability requirements. In cases with severe joint deformity, fibrous adhesion, or limited exposure, release of the LAAJ and, when necessary, C2 root sacrifice were performed to facilitate safe reduction and placement of interbody implants. All operations were performed by the same senior spine surgery team experienced in posterior reduction and fusion for atlantoaxial disorders. Routine postoperative follow-up examinations were conducted at 3, 6, and 12 months after surgery and annually thereafter. Follow-up assessment included clinical symptoms, VAS, neurological function, and radiological evaluation of reduction and fusion status when appropriate.

### Statistical analysis

Descriptive statistics were used, because of the small number of confirmed NTS cases and the exploratory nature of the study. Continuous variables are presented as mean ± standard deviation or median (range), and categorical variables as counts and percentages. Statistical analyses were performed using SPSS version 22.0 (SPSS Inc., Chicago, IL, USA). No inferential statistical testing was performed; therefore, no significance threshold was applied.

## Results

### Prevalence and baseline characteristics

Among the 176 patients with AAD treated with posterior reduction and instrumented fusion, eight screened positive for suspected NTS symptoms. Two of these were excluded because the symptoms occurred after cervical manipulation or trauma. The remaining six patients fulfilled the ICHD-3 diagnostic criteria for NTS, corresponding to an observed prevalence of 3.4% (6/176) in this surgical AAD cohort. The demographic, etiological, clinical, and preoperative classification data of the six included patients are summarized in Table 1. Five patients had craniovertebral deformity-related AAD, and one patient had C1 tumour-related AAD. According to Yin's classification, five patients were classified as reducible AAD (RAAD) and one as irreducible AAD (IAAD).

**Table 1:** Demographic, etiological, and preoperative clinical characteristics of patients with NTS secondary to AAD.

Case	Age (years)	Sex	Etiology	Preoperative AAD classification	NTS duration (months)	Main NTS features	Preoperative VAS	Preoperative Frankel grade
1	40	Female	Craniovertebral deformity	RAAD	6	Severe dysarthria/speech difficulty	6	B
2	22	Male	Craniovertebral deformity with whiplash-type neck injury	RAAD	12	Tongue deviation after whiplash-type neck injury; right-sided hemilingual numbness/discomfort triggered by prolonged speaking or prolonged sitting	4	E
3	14	Female	Craniovertebral deformity	IAAD	12	Tongue-shortening sensation and dysarthria triggered by prolonged speaking; gait instability	5	C
4	55	Female	Craniovertebral deformity	RAAD	17	Dysarthria	6	E
5	46	Male	C1 tumor-related AAD	RAAD	3	Severe head-neck and oropharyngeal pain, interpreted as possible tumor-related referred pain	8	E
6	39	Female	Craniovertebral deformity	RAAD	3	Tongue stiffness with occasional drooling	5	E

AAD, atlantoaxial dislocation; IAAD, irreducible atlantoaxial dislocation; NTS, neck-tongue syndrome; RAAD, reducible atlantoaxial dislocation; VAS, visual analogue scale.

### Clinical presentation

All six patients reported occipito-cervical pain and reproducible ipsilateral tongue symptoms provoked or aggravated by head turning, sustained speaking, prolonged sitting, or cervical posture. Symptoms included dysarthria, hemilingual numbness or discomfort, tongue stiffness, tongue deviation, tongue-shortening sensation, drooling, and oropharyngeal pain. Symptoms were alleviated by wearing a cervical collar or by complete relaxation of the cervical musculature in the supine position. Two patients exhibited mild hemilingual atrophy and varying degrees of dysarthria. Preoperative VAS scores ranged from 4 to 8 (mean 5.67 ± 1.37).

### Neurological status and imaging

Four patients experienced limb sensory disturbances. Frankel grades were B in one patient, C in one patient, and E in four patients preoperatively. All patients demonstrated imaging evidence of atlantoaxial malalignment and instability, with abnormal motion at the LAAJ and/or increased ADI on dynamic radiographs.

### Surgical details and follow-up outcomes

All six patients underwent posterior reduction and instrumented fusion of the atlantoaxial complex. C2 root sacrifice was required in two cases to allow adequate exposure, the bilateral arthrolysis and interarticular fusion. Follow-up ranged from 3 to 63 months (median 30 months). One patient died at 3 months due to tumor recurrence and metastasis. In the remaining patients, NTS-related pain and tongue symptoms improved after surgery: two experienced immediate relief, and four showed gradual improvement within 1–27 months.

**Figure 5:**
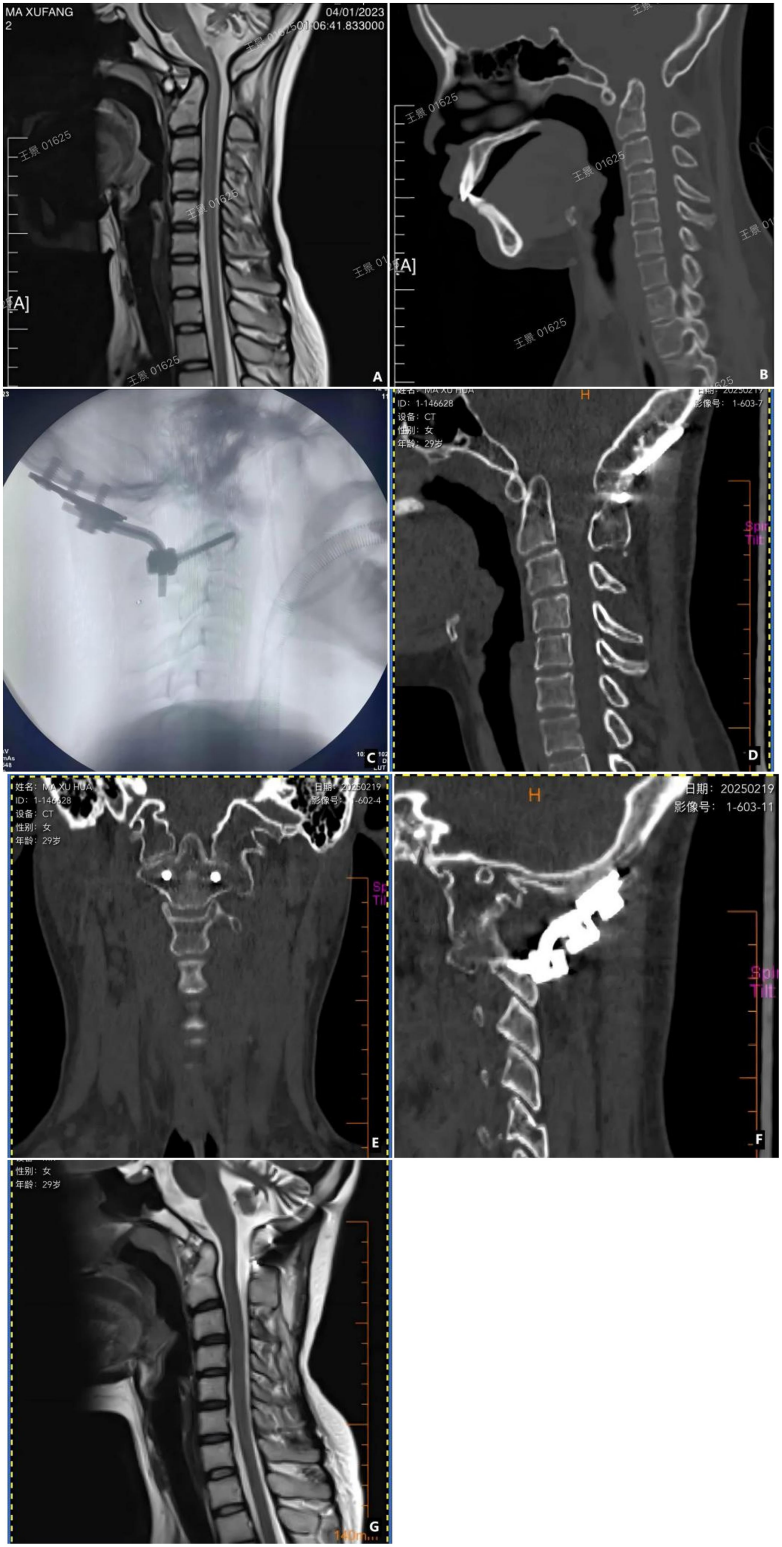
Representative case. The female patient presented with gait instability and a “tongue-shortening” sensation with dysarthria triggered by prolonged speaking for 6 months, and was diagnosed with AAD, NTS, and congenital occipito-cervical fusion. She underwent posterior reduction and occipito-cervical fusion. Gait instability improved postoperatively, and NTS symptoms completely resolved at 27-month follow-up. **(A)** Preoperative sagittal T2-weighted MRI showing separation at the atlanto-odontoid complex with posteriorly displaced odontoid compressing the spinal cord. **(B)** Preoperative sagittal 3D-CT reconstruction demonstrating atlantoaxial dislocation. **(C)** Intraoperative fluoroscopy during posterior reduction and occipito-cervical fusion. **(D)** Sagittal 3D-CT at 27 months showing satisfactory reduction of the atlantoaxial joint. **(E)** Coronal CT demonstrating osseous fusion across the LAAJ. **(F)** Sagittal 3D-CT showing interfacet fusion. **(G)** Follow-up MRI confirming reduction of the atlanto-odontoid complex and decompression of the spinal cord.

At the last follow-up, VAS scores decreased to 0–3 (mean 1.16 ± 1.47). Radiographs/ and CT confirmed satisfactory reduction (ADI < 3 mm) and solid fusion in surviving patients. Neurological function improved to Frankel D in two patients and Frankel E in four patients at the final assessment. Both patients who underwent C2 root sacrifice had persistent focal occipital/ or scalp sensory loss; no neurogenic ulceration or pressure sores were observed (Table 2, Figure 5).

**Table 2:** Surgical details and outcomes in patients with NTS secondary to AAD.

Case	Procedure level	Reduction technique/ cage use	C2 root sacrifice	Rationale for C2 root sacrifice	Complications	Preoperative VAS	Time to NTS symptom relief	VAS at last follow-up	Frankel grade at last follow-up	Radiographic reduction	Follow-up (months)
1	O-C3	Interfacet cages	Yes	Cage placement	Scalp numbness; no neurogenic ulcer	6	3 months	3	D	Yes	42
2	C1-C2	-	No	-	None reported	4	12 months	0	E	Yes	27
3	O-C2	-	No	-	None reported	5	27 months	1	D	Yes	30
4	C1-C2	-	Yes	Reduction/facilitation of exposure	Scalp numbness; no neurogenic ulcer	6	1 month	0	E	Yes	63
5	C1-C2	Interfacet cages	No	-	None reported	8	Immediate	3	E	Yes	3
6	C1-C2	-	No	-	None reported	5	Immediate	0	E	Yes	30

NTS, neck-tongue syndrome; O-C, occipitocervical; VAS, visual analogue scale.

All surgical procedures were performed by the same senior spine surgery team.

Radiographic reduction indicates satisfactory postoperative reduction confirmed by radiography and/or CT.

## Discussion

The purpose of this study was to characterize NTS secondary to AAD in a consecutive surgically treated cohort and to evaluate symptom response after posterior reduction and instrumented fusion. The main findings were as follows: NTS was identified in 3.4% of 176 surgically treated AAD patients; all six patients had reproducible tongue-related symptoms associated with neck movement, sustained speaking, or cervical posture; most cases were related to craniovertebral deformity; posterior reduction and instrumented fusion were associated with improvement of NTS-related pain and tongue symptoms; and C2 root sacrifice, when required for exposure or reduction, was followed by persistent focal occipital or scalp sensory loss but no neurogenic ulceration or pressure sores.

The observed prevalence of NTS in this surgical AAD cohort was higher than that reported in general population-based observations (2), although direct comparison is limited by referral patterns and case mix. This finding suggests that NTS may be under-recognised in patients with clinically significant AAD. Anatomically, the C2 root ventral ramus course close to the posterior aspect of the LAAJ and provide articular branches to the joint capsule. Prior anatomical work suggests that sensory fibers associated with tongue position sense may connect with this pathway near the LAAJ (10), offering a structural explanation for how irritation in this region can produce tongue symptoms.

Etiology and classification are important for interpreting the mechanism of symptoms. In the present series, five of six cases were related to craniovertebral deformity and one was related to C1 tumour-associated instability. According to Yin's classification, five patients had reducible AAD and one had irreducible AAD (12). These findings indicate that NTS may occur across different etiological backgrounds but may share a common mechanical pathway: abnormal motion, malalignment, or joint destruction at the atlantoaxial complex leading to repetitive irritation of the C2 neural structures.

AAD creates a setting in which such irritation is more likely to occur. The atlantoaxial lateral mass joints LAAJ contribute substantially to cervical rotation, and both congenital and acquired AAD are characterized by lateral mass joint instability (12, 13). During head rotation and flexion–extension, excessive translation at the LAAJ may repeatedly compress or stretch the nearby C2 nerve, which could trigger the combination of occipito-cervical pain and ipsilateral tongue sensory disturbance (1, 2, 5, 8, 14–17). Severity and chronicity may further amplify nerve irritation. Advanced AAD may be accompanied by osteophytes and remodeling at the LAAJ, broader joint destruction, and visible changes in the C2 root such as swelling or thickening. In some patients, changes in stabilizing ligaments,including Arnold's/accessory atlantoaxial ligament may tether the nerve near the LAAJ (18), increasing shear and traction during head movement. These factors may help explain why NTS appears in only a minority of AAD patients: individual differences in nerve course, surrounding soft-tissue cushioning, and how tongue-related sensory fibers join the C2 pathway may influence whether symptoms develop and how severe they become. Mild tongue atrophy in two patients may signal more sustained or severe involvement, potentially reflecting a broader pathological process in the atlantoaxial region (19).

The treatment response observed in this series supports the hypothesis that mechanical instability is central to AAD-associated NTS. In NTS related to minor trauma or manipulation, symptoms are often managed with medication cervical immobilization and physical therapy (7, 20–22). In contrast, conservative measures may be insufficient because the driver is mechanical instability. Our research outcomes, aligned with previous reports of operative management in selected patients (23), suggest that surgical stabilization can remove repetitive irritation at the LAAJ and thereby relieve symptoms

In severe AAD, sacrificing the C2 root may be necessary to safely achieve reduction and instrumentation, especially when adhesions or partial fusion across the LAAJ impede reduction and when an enlarged C2 root obstructs exposure (24). C2 root sacrifice can also immediately interrupt pain and sensory input transmitted through this pathway, which may explain rapid symptom relief in some cases. The previous report described partial improvement after C2 root resection in long-standing NTS associated with basilar invagination (23). In our two patients who underwent C2 root sacrifice, postoperative symptom relief may reflect restored atlantoaxial stability, interruption of C2 afferent input, or both. Nevertheless, C2 root sacrifice carries irreversible morbidity, including permanent focal scalp numbness and potential complications such as pressure-related scalp ulceration (25). In our group, no neurogenic ulceration or pressure sores occurred. This maneuver should therefore be reserved for selected cases with careful counselling and a clear risk–benefit rationale. Other craniovertebral abnormalities, such as Chiari-1 malformation, have also been reported in association with NTS (26), supporting careful evaluation of craniovertebral junction pathology in patients with secondary NTS.

### Limitations and future directions

Several limitations should be acknowledged. First, this was a retrospective single-center study with a small number of confirmed NTS cases, which limits statistical power and generalizability. Second, although we attempted to minimize under-ascertainment by systematically contacting all 176 patients and using a structured symptom interview with independent adjudication, transient symptoms are prone to recall bias, and some mild episodes may still have been forgotten or misattributed. Third, because the main outcome was based on clinical symptom resolution, objective neurophysiological confirmation of the afferent pathway was not available. Fourth, because of the retrospective design, standardized health-related quality-of-life (HRQoL) questionnaires, such as the Neck Disability Index (NDI) or other validated patient-reported outcome measures (PROMs), were not systematically collected in all patients, especially in earlier cases, therefore, functional recovery and HRQoL could not be evaluated uniformly. Larger multicenter prospective studies with standardized symptom capture, validated PROMs, and longer follow-up are warranted to validate the proposed mechanism and treatment outcomes.

## Conclusions

AAD should be recognised as an important aetiology of secondary NTS. Dynamic atlantoaxial instability, particularly at the LAAJ, may repetitively irritate the adjacent C2 spinal nerve/ventral ramus and provoke the characteristic combination of occipito-cervical pain and ipsilateral hemilingual sensory disturbance. Posterior reduction and instrumented fusion can stabilise the atlantoaxial complex and are associated with substantial symptom improvement.

## Data Availability

The raw data supporting the conclusions of this article will be made available by the authors, without undue reservation.
